# Transplant-associated penile Kaposi sarcoma managed with single agent paclitaxel chemotherapy: a case report

**DOI:** 10.1186/s12894-021-00855-y

**Published:** 2021-06-07

**Authors:** Matthew A. Anderson, Tracey Ying, Kate Wyburn, Peter M. Ferguson, Madeleine C. Strach, Peter Grimison, Steve Chadban, David M. Gracey

**Affiliations:** 1 Department of Renal Medicine and Renal Transplant, Royal Prince Alfred Hospital, Missenden Rd, Camperdown, NSW Australia; 2 Kidney Node, Charles Perkins Centre, University of Sydney, Camperdown, NSW Australia; 3 Central Clinical School, Faculty of Medicine, University of Sydney, Camperdown, NSW Australia; 4 Department of Tissue Pathology, Royal Prince Alfred Hospital, Missenden Rd, Camperdown, NSW Australia; 5 Department of Medical Oncology, Chris O’Brien Lifehouse, Camperdown, NSW Australia

**Keywords:** Kaposi sarcoma, Transplant, Renal, Malignancy, Therapy, Chemotherapy, Immunosuppression, Sirolimus

## Abstract

**Background:**

Kaposi’s sarcoma is an uncommon complication in renal transplant patients, and typically presents with cutaneous lesions on the lower extremities. Penile involvement has been reported only rarely. Management of cutaneous-limited disease is primarily reduction of immunosuppression and conversion to an mTOR-inhibitor, whereas the treatment of disseminated disease in transplant patients is more variable.

**Case presentation:**

A 75-year-old male, originally from Somalia, received a deceased-donor kidney transplant for diabetic and hypertensive nephropathy. Seven months post-transplant he presented with lower limb lesions, oedema and bilateral deep vein thromboses. He then developed a fast-growing painful lesion on his penile shaft. A biopsy of this lesion confirmed KS, and a PET scan demonstrated disseminated disease in the lower extremities, penis and thoracic lymph nodes. His tacrolimus was converted to sirolimus, and his other immunosuppression was reduced. He was treated with single agent paclitaxel chemotherapy in view of his rapidly progressing, widespread disease. The penile lesion completely resolved, and the lower extremity lesions regressed significantly. His kidney allograft function remained stable throughout treatment.

**Conclusion:**

This case illustrates a rare presentation of an uncommon post-transplant complication and highlights the need for a high index of suspicion of KS in transplant patients presenting with atypical cutaneous lesions. It serves to demonstrate that the use of single agent paclitaxel chemotherapy, switch to an mTORi and reduction in immunosuppression where possible produces excellent short-term outcomes, adding to the body of evidence for this management strategy in disseminated Kaposi’s sarcoma.

## Background

Kaposi Sarcoma (KS) is a low-grade multi-centric tumour composed of endothelium-lined vascular spaces and spindle cells [1]. It was initially described in 1872 by Moritz Kaposi, and remained an uncommon phenomenon until 1979, when it was first recognised in solid organ transplant patients [2]. The incidence in kidney transplant recipients has since been reported to be as high as 500-times that of the general population [3]. Its pathogenesis derives from reactivation of human herpesvirus-8 (HHV-8) in the setting of immunosuppression. KS classically presents as angiomatous cutaneous lesions on the lower limbs. Disseminated disease with visceral involvement is uncommon and carries a poorer prognosis. The optimal management of disseminated disease in the transplant setting is not well defined.

## Case presentation

The case was a 75-year-old male of Somali heritage, who received a deceased donor kidney transplant for presumed diabetic and hypertensive nephropathy. The donor was marginal but immunologically low-risk, being a 4/6 HLA mismatch with a single weak class II donor-specific antibody. At the time of transplant, he received our unit’s standard induction immunosuppression with basiliximab and methylprednisolone, then commenced maintenance immunosuppression with tacrolimus, everolimus, and prednisone. A denovo mTOR inhibitor (mTORi) was chosen in place of mycophenolate mofetil due to the recipients older age, pre-existing leukopenia and known vascular disease in the donor. The transplant was complicated by delayed graft function necessitating haemodialysis on day one. A renal biopsy on day nine demonstrated borderline acute T-cell mediated rejection; as such, the everolimus was replaced with mycophenolate mofetil. The graft function subsequently improved to a baseline creatinine of 170 µmol/L (eGFR 34 ml/min) with minimal proteinuria (albumin: creatinine ratio 2.6 mg/mmol). A protocol biopsy at eight weeks showed no features of rejection.

**Fig. 1:**
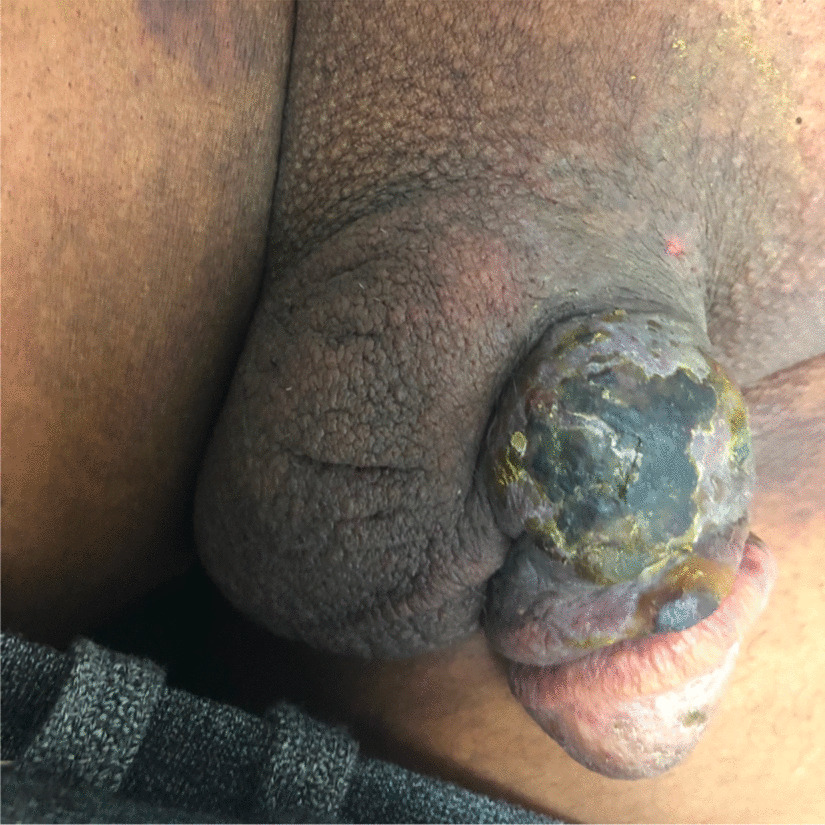
Painful rapidly growing penile lesion in a renal allograft recipient 7 months post-transplant

Seven months post-transplant the recipient presented with bilateral lower limb pain and swelling, and dark macular skin lesions on his legs. He underwent a duplex ultrasound which demonstrated bilateral proximal deep vein thromboses. Anticoagulation with apixaban was commenced. Soon after he presented with a painful papular lesion on his penile shaft, which began as a small macule and had grown rapidly (Fig. 1). There was no associated exudate or surrounding erythema, nor associated urinary symptoms. Two small satellite lesions had also erupted on the glans of the penis. The patient was referred for a biopsy.

**Fig. 2:**
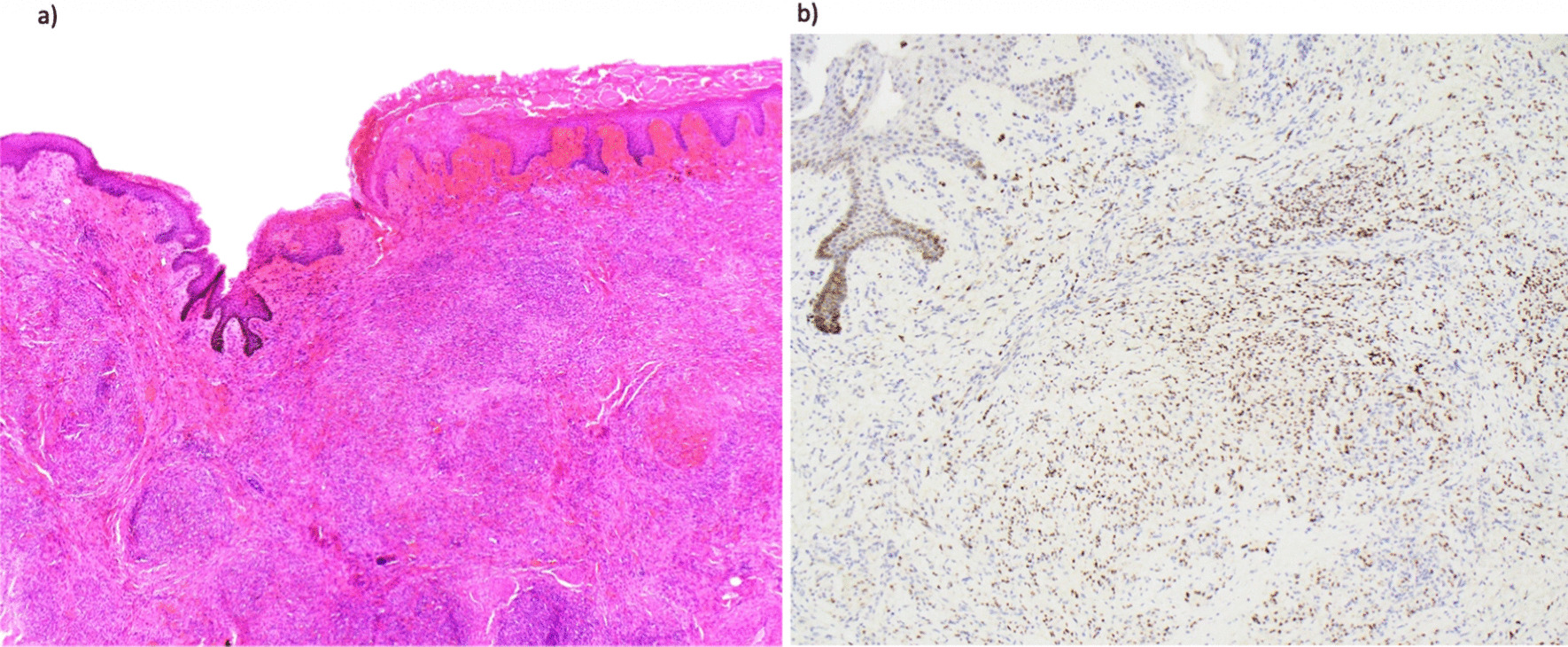
Biopsy of penile lesion biopsy demonstrating: **a** diffuse infiltration of the stroma by spindled cells forming slit-like anastomosing spaces containing erythrocytes (40× magnification), and **b** immunohistochemistry staining for human herpesvirus-8 (HHV-8) (100× magnification)

A biopsy of the penile lesion demonstrated diffuse involvement of the dermis by nodules of mitotically active spindled cells, with irregular slit-like spaces, which stained positive for HHV-8, diagnostic of KS (Fig. 2).

**Fig. 3:**
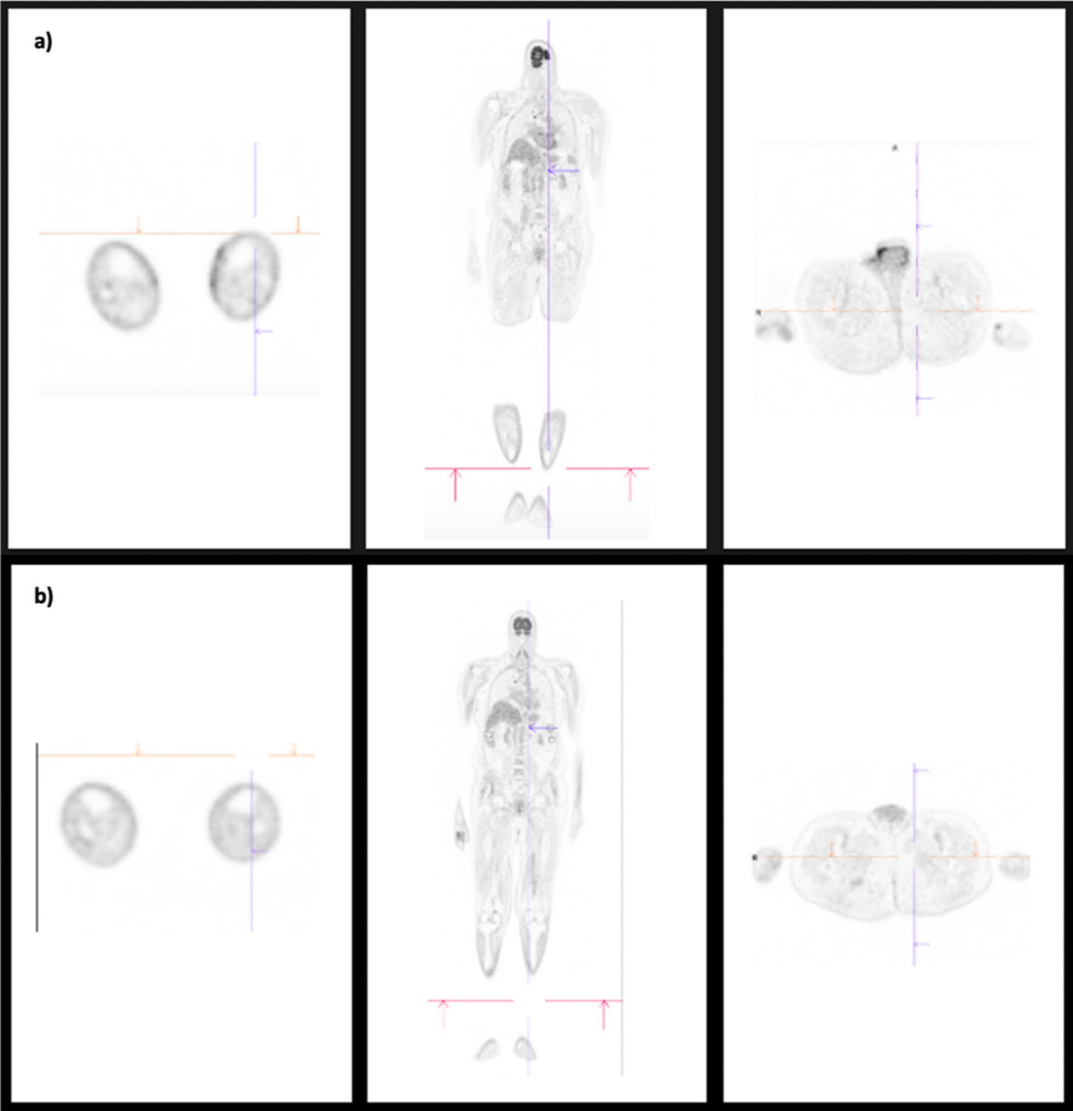
**a** PET scan at diagnosis demonstrating diffusely increased metabolism in the skin and subcutaneous tissues of the feet, lower legs and over the surface of the penis, as well as abnormally increased metabolism in the paratracheal lymph nodes (middle image, arrow). **b** PET scan after three months of therapy demonstrating decreased metabolism of the skin and subcutaneous tissues of the lower limbs, absence of previously identified increased metabolism in the penile shaft, and reduced but ongoing increased metabolism in the right paratracheal node (arrow)

A PET scan demonstrated diffusely increased metabolism in the skin and subcutaneous tissues of the feet and lower legs, and over the surface of the penis. There was also abnormally increased metabolism in the paratracheal lymph nodes, with no increased metabolism in the inguinal, femoral, pelvic or abdominal nodes nor visceral organs (Fig. 3a).

In view of the rapidly progressive disseminated disease, the recipient commenced weekly single-agent paclitaxel chemotherapy with a plan for twelve weeks in total. His immunosuppression was reduced, with the mycophenolate ceased, prednisone dose tapered to 5 mg daily, and the tacrolimus switched to sirolimus, with a dose of 1.5 mg daily maintaining a level of 5-8 µg/L.

The recipient responded well to chemotherapy and reduced immunosuppression. After four weeks of paclitaxel the penile lesion resolved completely with no residual scarring, and the lower limb lesions significantly regressed. A progress PET scan (Fig. 3b) demonstrated decreased metabolism of the skin and subcutaneous tissues of the lower limbs, and absence of previously identified increased metabolism in the penile shaft. There was ongoing increased metabolism in the right paratracheal node. The only complication of chemotherapy was a grade 1 peripheral neuropathy. On most recent review, 11 months following the initial presentation, there has been no recurrence of Kaposi sarcoma.

Throughout the treatment the patient’s transplant function remained stable, with no evidence of rejection despite the reduction in immunosuppression. He is now 18 months post-transplant, with the most recent pathology demonstrating a creatinine of 162 µmol/L, with no proteinuria (ACR 1.3 mg/mmol).

## Discussion and conclusions

Solid organ transplant is associated with an increased risk of de novo malignancy, with registry data demonstrating an incidence up to fourfold higher than the general population [4] [5] [6]**.** Immunosuppression is hypothesised to have a carcinogenic effect through impaired immunosurveillance mechanisms and direct damage to host DNA, as well as the potentiation of proto-oncogenic viruses [7].Of all post-transplant malignancies, KS has by far the highest standardised incidence ratio compared to the general population, with reported ratios ranging from 22 in the ANZDATA registry [8] to 61 in US data [9].

Four distinct clinical subtypes of KS are described: classic; organ-transplant associated; epidemic (AIDS-related); and endemic (a unique aggressive subtype that occurs in equatorial African patients without HIV).

HHV-8 has been causally associated with all forms of KS [10]. The seroprevalence of HHV8 in population studies is highest in the Mediterranean and Central Africa [11, 12]. Paralleling this, transplant recipients of African and Mediterranean origin are consistently identified to have the highest rates of KS post-transplant [9].

In more than 90% of cases, KS presents as angiomatous cutaneous lesions in the lower extremities. Associated lymphedema is common, and often precedes the appearance of cutaneous lesions. Disseminated visceral involvement occurs in approximately 25% of kidney transplant-associated KS, with higher rates seen in liver and heart transplant recipients [13] [14]. The most frequently implicated extra-cutaneous sites are the lymph nodes, gastrointestinal tract and lungs.

Genital KS is rare and reported almost exclusively in AIDS-associated KS, where the incidence is approximately 3% [15]. Only a single case report of penile KS in a transplant patient is reported in the literature [16]. In that case, the KS was cutaneous-limited (confined to the glans of the penis), and the patient was treated with reduction in immunosuppression and conversion to mTORi; however, they sustained subsequent graft loss due to acute rejection.

Reduction in immunosuppression is the cornerstone of management in transplant-associated KS and is often adequate in treating cutaneous-limited disease. However, any reduction in immunosuppression must be balanced with the risk of allograft loss from rejection. Switching the more oncogenic immunosuppressants (particularly calcineuirin inhibitors) for an mTORi has become a commonly accepted management strategy. The efficacy of this approach was demonstrated in a 2005 case series of 15 patients, where cessation of cyclosporine and commencement of sirolimus was associated with complete resolution of cutaneous KS in all patients, with no reports of graft dysfunction [1].

The management of disseminated KS in transplant recipients is not well described in the literature. In 2019 a retrospective analysis was published describing the management strategies of 145 organ transplant recipients with KS across different European centres [17]. Of these patients, 51% had disseminated disease at diagnosis. Their first-line management was highly variable, with 95% of patients having a reduction in immunosuppression, 28% being converted to an mTORi, and 16% receiving chemotherapy [14 anthracycline, 1 paclitaxel] in addition to immunosuppression regimen alteration. Patients with disseminated disease who received conversion to an mTORi and short-term chemotherapy had the best response rates.

There are no guidelines recommending which chemotherapeutic agent to use in disseminated transplant-associated KS. Treatment decisions are typically based off safety and efficacy data extrapolated from AIDS-related and classic KS cohorts with disseminated disease. In a 2020 update on therapy for Kaposi sarcoma, bleomycin and vinblastine were identified as the most widely employed single-agent therapies [18]. However, they are often poorly tolerated, particularly in elderly patients, and their use is limited by potential toxicity. Taxanes (paclitaxel and docetaxel) and liposomal doxorubicin are better tolerated and have been found to be efficacious in disseminated KS, and thus are being used more often in this setting [19, 20]. There are also reports of responses to anti-programmed-cell-death-protein immune checkpoint inhibitors, however their safety in renal transplant recipients has not been established [21].

Management of KS in transplant recipients is particularly complex due to the risk of rejection and graft loss with immunosuppression reduction, and the increased risk of infection when already immunosuppressed patients are treated with chemotherapy. Outcomes for disseminated disease have traditionally been poor. In 1997 Penn reported a mortality rate of 57% in those with disseminated disease (22). In a 2005 South African retrospective analysis of 21 transplant patients with KS, six had disseminated disease, three died rapidly, while the other three survived but suffered allograft loss due to acute rejection.

In summary, we present a case of a very rare manifestation of KS in a kidney transplant recipient. Penile lesions in immunosuppressed men should prompt consideration for KS and early referral for biopsy. Given the rarity of this disease, trials in this area are not feasible. There are no clear guidelines for optimal management of disseminated KS in transplant patients, and prognosis for allograft function and mortality are poor. This case serves to demonstrate that treatment with single agent taxane chemotherapy, switch to an mTORi, and reduction in immunosuppression where possible produced excellent short-term outcomes, and thus should be considered as a potential management strategy in similar patients.

## Data Availability

Not applicable.

## Abbreviations

KS: Kaposi sarcoma
HHV-8: Human herpesvirus 8
HLA: Human leukocyte antigen
mTOR: Mammalian target of rapamycin
mTORi: Mammalian target of rapamycin inhibitor
eGFR: Estimated glomerular filtration rate
ACR: Albumin:creatinine ratio
DVT: Deep vein thrombosis
PET: Positron emission tomography
ANZDATA: Australian and New Zealand Dialysis and Transplant Registry
AIDS: Acquired immunodeficiency syndrome
HIV: Human immunodeficiency virus
